# The mismatch repair endonuclease MutLα tethers duplex regions of DNA together and relieves DNA torsional tension

**DOI:** 10.1093/nar/gkad096

**Published:** 2023-02-25

**Authors:** Scott J Witte, Isabella M Rosa, Bryce W Collingwood, Jonathan M Piscitelli, Carol M Manhart

**Affiliations:** Department of Chemistry, Temple University, Philadelphia, PA 19122, USA; Department of Chemistry, Temple University, Philadelphia, PA 19122, USA; Department of Chemistry, Temple University, Philadelphia, PA 19122, USA; Department of Chemistry, Temple University, Philadelphia, PA 19122, USA; Department of Chemistry, Temple University, Philadelphia, PA 19122, USA

## Abstract

In eukaryotic mismatch repair, MutS homologs recognize mismatches and recruit the MutLα endonuclease which introduces a nick in the newly replicated, error-containing DNA strand. The nick occurs in response to the mismatch, but at a site up to several hundred base pairs away. The MutLα nick promotes mismatch excision by an exonuclease (Exo1) or removal by the strand displacement activity of a DNA polymerase which may work in conjunction with a flap endonuclease. Models have suggested that MutL homolog endonucleases form oligomeric complexes which facilitate and are activated by strand capture mechanisms, although such models have never been explicitly tested. We present evidence that the mismatch repair MutLα endonuclease is activated by DNA–DNA associations and that it can use this property to overcome DNA torsional barriers. Using DNA ligation and pull-down experiments, we determined that the MutLα endonuclease associates two DNA duplexes. Using nuclease assays, we determined that this activity stimulates MutLα’s endonuclease function. We also observe that MutLα enhances a topoisomerase without nicking the DNA itself. Our data provide a mechanistic explanation for how MutL proteins interact with DNA during mismatch repair, and how MutL homologs participate in other processes, such as recombination and trinucleotide repeat expansions.

## INTRODUCTION

DNA mismatch repair is a highly conserved pathway that maintains genomic integrity and functions to repair mis-incorporated bases during DNA replication processes. Defects in genes encoding mismatch repair proteins are associated with Lynch syndrome, an inherited cancer syndrome associated primarily with a predisposition to colorectal and endometrial cancers in addition to sporadic tumors (1).

In the major eukaryotic mismatch repair pathway, DNA mismatches are first recognized by MutSα (Msh2–Msh6) or MutSβ (Msh2–Msh3) proteins, which then recruit the MutLα (Mlh1-Pms1 in yeast, MLH1-PMS2 in mouse and human) endonuclease (2). After activation by the replication processivity factor, proliferating cell nuclear antigen (PCNA), MutLα hydrolyzes, or nicks DNA within a few hundred base pairs from the mismatch (3–5). MutLα-generated nicks 5′ to the mismatch are used to remove the lesion via excision by a 5′ to 3′ exonuclease (Exo1). The resultant gap is bound by RPA, filled by DNA polymerases δ and ϵ, and sealed by DNA ligases (2,6). An Exo1-independent pathway exists where MutLα makes multiple nicks on the nascent strand near the mismatch. DNA polymerase δ then removes the mismatch via strand displacement, which also initiates on the 5′ side of the mismatch, with flaps being removed by the Rad27 flap endonuclease (7–10). Models using reconstituted systems demonstrate that although MutLα nicks DNA in response to a mismatch detected by a MutS homolog, the endonuclease acts at a distant site yet is bounded by the position of the mismatch (3,4,11). Mechanisms for how MutLα endonuclease activity is distantly constrained by a mismatch in DNA mismatch repair are not well established.

Although a population of MutSα tracks with the replication machinery, MutLα does not colocalize with replication forks (12). This suggests that MutLα interacts with DNA some distance behind the replisome. DNA in this region is not yet packaged into nucleosomes and may adopt higher-ordered, tertiary structure which the downstream DNA mismatch repair proteins, such as MutLα, PCNA, and excision factors may need to navigate in order to locate a MutSα-bound mismatch. How MutLα nicking is restrained to a region of DNA near the mismatch and how repair proteins downstream of mismatch recognition interact with damaged DNA in a potentially topologically complex environment is not well understood.

Past studies have elucidated some mechanistic details for how MutLα interacts with DNA. Work by Hall *et al.* has shown that MutLα binds DNA non-specifically, primarily through electrostatic interactions in a cooperative manner by showing that the protein has higher affinity for larger DNA molecules compared to smaller DNA molecules (13). This trend was also observed for the yeast meiotic homolog MutLγ and human MutLα in relation to the two proteins’ endonuclease activities (14). Together, these data have been used to propose models for MutL homologs that involve large oligomeric complex formation and that polymers of MutL proteins must reach a critical length in order to support endonuclease activity. Larger DNA substrates are preferred relative to smaller substrates because they provide a longer expanse of DNA on which to accommodate a MutL homolog oligomer of sufficient length to promote endonuclease activity. Additionally, yeast MutLα has been shown to bind to circular DNA substrates with higher affinity than equivalent size and sequence substrates linearized with a restriction enzyme (13). This trend was also shown for the endonuclease activity of human MutLα and yeast MutLγ (14). These observations are consistent with MutL homolog oligomers in that on circular DNA, all protein binding sites are equivalent and initial binding will support polymerization in any direction. A MutL homolog polymer of sufficient size to activate the endonuclease can form provided that the circular DNA is large enough to support the activity. On linear DNA, initial binding near an end could promote the protein to polymerize toward the end and ultimately slide off, accounting for the lower activity on these substrates. MutL homolog oligomers are also described by work investigating the recruitment and activation of MutLα by MutSα in the human system. In these experiments, an excess of MutLα was shown to be present relative to MutSα using sucrose gradient fractionation (15). Similarly, multiple studies have also observed a requirement of a stoichiometric excess of MutL to MutS protein in reconstituted and partially reconstituted bacterial, yeast and human systems (4,16–20). These data suggest that MutL homolog oligomers are necessary for endonuclease activation, but they may also serve other roles, as well. This is proposed because work in *Escherichia coli* also supports a role for MutL homolog polymers even though *E. coli* MutL is not an endonuclease. In DNase I footprinting experiments using the *E. coli* proteins, a large region of mismatched DNA becomes protected from digestion only after recruitment of MutL by MutS, suggesting that multiple MutL proteins are present on the model substrate (21). Despite the abundance of evidence for MutL homolog polymers, a complete functional role for this property in mismatch repair is not well established.

To directly visualize a MutL homolog oligomer and to potentially shed light on its mechanistic roles, Hall *et al.* used atomic force microscopy to visualize long tracts of MutLα on plasmid substrate (13). The authors observed that a subset of the MutLα tracts appeared to condense two duplex regions of DNA, suggesting that a role for MutL homolog oligomers may be to promote DNA–DNA associations. Although the ability of the oligomeric complex to interact simultaneously with two DNA molecules was not explicitly tested, recent biophysical work further supports MutLα’s putative ability to condense duplex DNA (18). Further supporting a potential DNA tethering activity for MutL homolog oligomers is work showing that yeast MutLγ could nick a small linear substrate, that was not nicked in isolation, in the presence of a larger circular substrate that is efficiently nicked (14). In aggregate, these data potentially point to a strand capture model where DNA–DNA associations are driven by large MutL homolog nucleoprotein complexes, the formation of which, is also necessary to activate the endonuclease function. This model has not been tested or measured, however, and a role for this activity in mismatch repair is also unclear.

Here, we provide evidence to suggest that *Saccharomyces cerevisiae* MutLα polymeric complexes tether duplex regions of DNA and this property stimulates the protein's latent nuclease activity. We also demonstrate that MutLα can enhance conversion of supercoiled to relaxed DNA by a topoisomerase in the absence of PCNA and the MutLα endonuclease activity. This activity could be used to position the region of duplex DNA that is nicked adjacent to the endonuclease active site. These data can explain how MutLα endonuclease activity is restrained to an area by the mismatch in DNA mismatch repair and how the protein may overcome torsional barriers behind the replication fork. These data also support models for how MutL homologs participate in meiotic recombination which involves substrate with adjacent DNA duplex regions.

## MATERIALS AND METHODS

### Purified protein and DNA substrates used this study

Yeast MutLα was expressed and purified from *S. cerevisiae* using previously reported methods (22). Yeast RFC and PCNA were expressed and purified from *E. coli* according to previously published methods (23,24).

Unless otherwise noted, 2.7 kb and 4.3 kb closed circular substrates are commercially purchased 2.7 kb pUC18 and 4.3 kb pBR322 plasmids from Invitrogen.

### Gel imaging and quantifications

All agarose gels were stained by soaking in 1× TAE (Tris-acetate-EDTA) buffer containing 0.66 μg/ml ethidium bromide unless otherwise indicated. Gels were imaged on a Sapphire Biomolecular Imager (Azure) and quantified using ImageJ software (NIH).

### DNA ligation assays

Linear DNA fragments with overhangs were generated by incubating 4.9 μg of 2.7 kb pUC18 with 100 units of HindIII (5′-overhangs), SphI (3′-overhangs), BsaI (5′ overhangs) according to the manufacturer's (NEB) instructions followed by a heat inactivation step.

20 μl ligation reactions were assembled on ice in Buffer A, which contains 25 mM HEPES pH 7.5, 40 μg/ml of BSA, 1 mM DTT, 25 mM NaCl and 3% glycerol (final concentrations). 3.8 nM DNA was combined with 0.25 mM ATP, and the indicated concentration of MutLα. For conditions that promoted the reformation of circular DNA, the final concentration of DNA was 1.4 nM. Reactions were incubated at 22°C for 10 min, followed by the addition of 0.8 units of T4 DNA ligase (NEB) in a second step. Reactions were then incubated for 1 h at 22°C. Reactions were stopped by incubation in stop mix (final concentrations: 1% SDS, 14 mM EDTA, 0.96 units proteinase K) at 37°C for 15 min to denature and digest the protein component of the reaction. Reaction products were resolved by 0.7% (w/v) agarose gel run at 100 V for 45 min in 1× TAE, followed by ethidium bromide staining.

### DNA pull-down experiments

4.3 kb pBR322 was biotinylated with psoralen-PEO biotin (Pierce) according to the manufacturer's instructions. Briefly, 10 μg of plasmid was denatured for 5 min at 99°C then combined with the psoralen-PEO biotin reagent at a final concentration of 200 μM. The sample was then irradiated for 30 min with UV light (350 nm) in a Rayonet Photochemical Reactor to attach the biotin moieties to the DNA.

For pull-down experiments, ∼150 ng of biotinylated pBR322 was immobilized on 0.3 mg of magnetic streptavidin beads (Thermo) and washed with Buffer A to remove unbound DNA (for experiments containing ATPγS, 0.25 mM was added immediately following wash with Buffer A). MutLα was then incubated with the immobilized DNA in Buffer A for 10 min at 22°C. The beads were magnetically pulled down and washed with 20 μl of Buffer A. Unlabeled 2.7 kb pUC18 (∼135 ng) was then added and incubated at 22°C for 10 min, followed by three washes with Buffer A (20 μl each wash). Proteinase K (0.96 units) was then added to digest MutLα to determine if the MutLα bound to biotinylated 4.3 kb plasmid pulled down 2.7 kb unlabeled plasmid. The supernatant and bead-bound material was analyzed by 1.2% (w/v) agarose gel run and stained with ethidium bromide. Bead-bound fractions were incubated at 95°C for 5 min prior to gel loading for both gel systems.

Duplicate experiments to those above were analyzed by 8% SDS-PAGE. Samples from each fraction (10 μl) were combined with 5 μl of 3× sample buffer (final concentrations: 60 mM Tris pH 6.8, 1% SDS, 1% glycerol, 0.33% bromophenol blue and 5% 2-mercaptoethanol) and boiled at 95°C for 5 min prior to resolving by SDS-PAGE. Gels were fixed in a solution of 40% ethanol and 10% acetic acid and stained in 1× Flamingo gel strain (BioRad) according to the manufacturer's instructions.

### Endonuclease assays

Endonuclease reactions were performed as previously described (25,26). Briefly, 20 μl reactions were combined in a buffer containing 20 mM HEPES–KOH, 20 mM KCl, 1% glycerol, 0.2 mg/ml BSA, 2.5 mM MnSO_4_ and 0.25 mM ATP (final concentrations). Reactions were incubated at 37°C for 60 min and stopped using stop mix as described above. Endonuclease product was resolved by 1.2% (w/v) agarose gel run in 1× TAE as described for ligation assays or by denaturing 1% (w/v) agarose gel containing 30 mM NaCl and 2 mM EDTA run in solution containing 30 mM NaOH and 2 mM EDTA. Prior to sample loading, solution containing 30 mM NaOH, 1 mM EDTA, 3% glycerol, and 0.02% bromophenol blue (final concentrations) were added to the reactions, the mixtures were heated at 70°C for 5 min and cooled for 3 min on ice. Denaturing gels were resolved at 50 V for ∼3 h. Gels were neutralized in 0.5 M Tris buffer (pH 7.5) for 30 min followed by staining as described above (14,25). For gels run in 1× TAE, negative control lanes were used as background and subtracted from quantifications. For denaturing gels, endonuclease activity was measured as amount of DNA lost relative to negative control lanes.

### Topoisomerase assays

Topoisomerase assays were performed using commercially available *E. coli* Topoisomerase I (NEB). 20 μl reactions containing 3.8 nM 2.7 kb supercoiled pUC18 DNA (Thermo) and varying concentrations of MutLα were combined on ice in buffer containing 50 mM potassium acetate, 20 mM Tris-acetate, 10 mM magnesium acetate, 100 mg/ml BSA and 1 mM DTT (final concentrations) at pH 7.9. Reactions were incubated for 10 min at 22°C. After the initial incubation, 0.25 units of Topoisomerase I were added and incubated for 20 min at 37°C. Reactions were stopped using stop mix as described above, resolved by 1.2% agarose gel for 1 hour at 100 volts, and stained as described above (14,25).

Some figures modelling MutLα behaviors were made in part with BioRender.

## RESULTS

### MutL*α* stimulates DNA associations

Although MutL protein oligomers have been detected in multiple systems, their functional relevance is not established. Atomic force microscopy and negative stain electron microscopy experiments have been used to visually detect large MutL protein complexes and have shown that formation of a MutL oligomer is dependent on DNA, which is consistent with the cooperativity measured in DNA binding assays (13,14). This suggests that the protein is behaving similarly in static microscopy experiments and dynamic experiments in solution. Atomic force microscopy experiments have also shown that when multiple yeast MutLα proteins are bound to DNA, the DNA becomes condensed (13,18), but the nature of this compaction, its functional relevance, and how it facilitates mismatch repair is not understood. For the meiotic homolog MutLγ, DNA strand capture or associations between MutLγ-bound DNA duplexes have been proposed to promote endonuclease activity which may provide a clue to the mechanism of MutL homolog endonucleases (14). Despite proposals for these behaviors, it has not been shown, however, whether MutLα complexes can tether DNA duplexes together in solution. Moreover, a functional role for these complexes and whether such an activity is required to activate the endonuclease is also unestablished.

**Figure 1.**
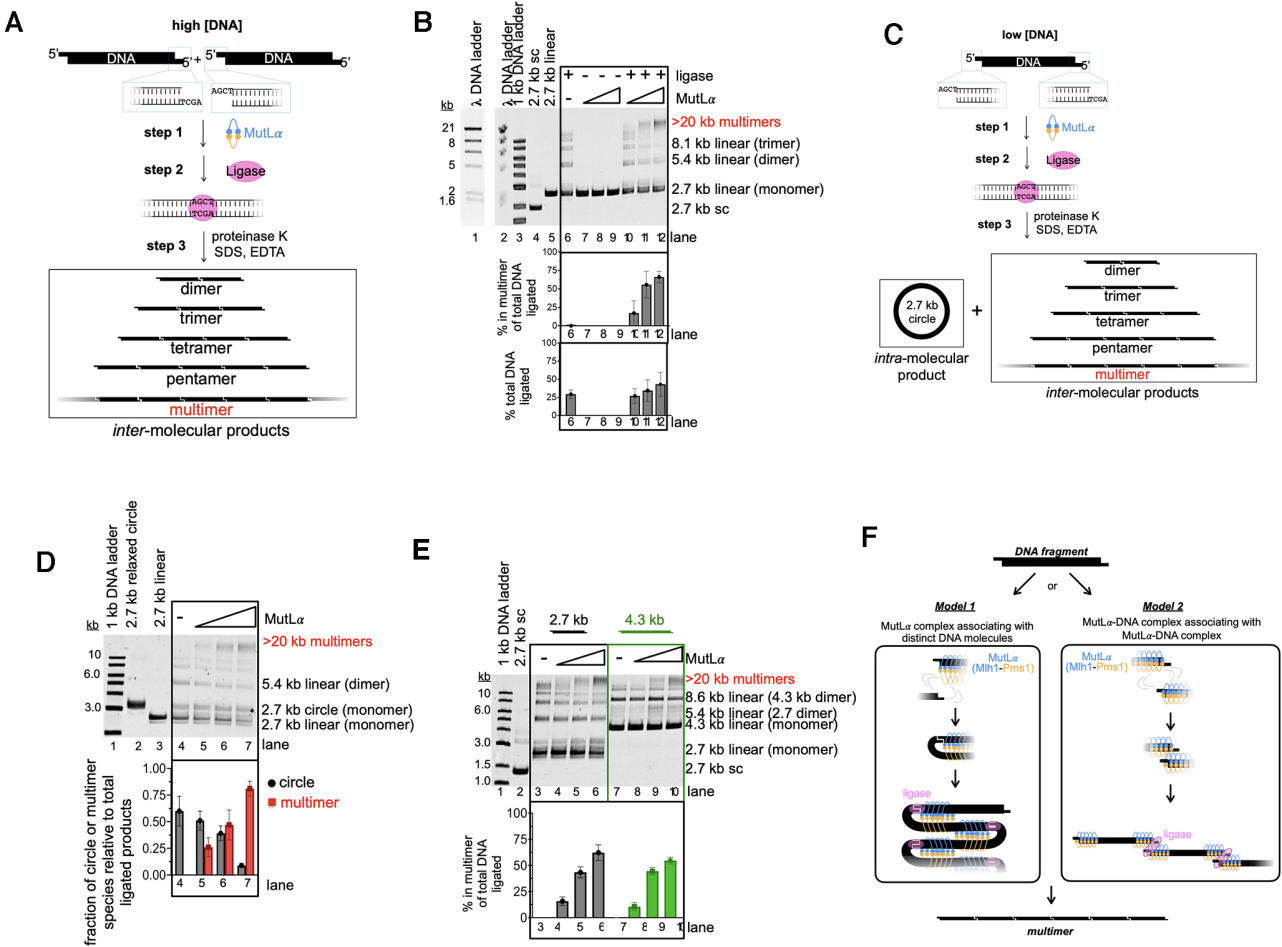
MutLα complexes facilitate inter-molecular associations. (**A**) Schematic depicting ligation assays. 2.7 kb DNA is linearized with a restriction enzyme, creating ligatable ends. T4 DNA ligase is added at low concentrations so that inter-molecular products are preferentially created after MutLα is bound to DNA where indicated. (**B**) Ligation experiment using linearized 2.7 kb DNA with 5′ overhangs generated by HindIII restriction digest. Lanes 1 and 2 contain DNA markers generated by digesting lambda DNA (NEB) with HindIII. Lane 3 contains 1 kb ladder (NEB). Lanes 4 and 5 contain covalently closed, supercoiled (sc) circular 2.7 kb DNA and linear DNA starting material, respectively. Where +, T4 DNA ligase is added as indicated in the Materials and Methods. In lanes where a titration of MutLα is indicated the concentration of protein is 50, 150 and 300 nM. The amount of signal in the highest molecular weight band was quantified relative to the total amount of ligated product in each lane and the total amount of DNA ligated was quantified relative to total amount of DNA in lane. The average values for these are expressed in bar graph below the gel. The experiment was performed in triplicate and error bars represent standard deviations between experiments. (**C**) Schematic depicting ligation assay using low DNA concentrations where reformation of the circular intra-molecular product is preferred. (**D**) Top, Ligation assay using low DNA concentration (1.4 nM relative to 3.8 nM used in panels B and D). MutLα was titrated and lanes 5–7 contain 50, 150 and 300 nM, respectively. Bottom, graph depicts the amounts of DNA in each product relative to the total amount ligated. Number of replicates is *n* = 3. (**E**) Ligation assay using linearized 2.7 and 4.3 kb DNA with 5′-overhangs generated by HindIII. Lanes 4–6 and 8–10 are titrations containing 50, 150 and 300 nM MutLα.Number of replicates is n = 3. (**F**) Models for MutLα forming DNA multimers by promoting inter-molecular associations.

To determine if MutLα can enhance *inter-*molecular DNA–DNA associations, we created 2.7 kb linear substrates with ligatable four nucleotide 5′-overhangs and identified conditions where T4 DNA ligase favors *inter*-molecular ligation products (Figure 1A, B). We took advantage of MutLα’s ability to bind to DNA ends (27) and conditions that do not support MutLα endonuclease activity (i.e.—in the absence of the activating factor PCNA), and titrated MutLα into ligation reactions. In the presence of MutLα and T4 DNA ligase, we observed a high molecular weight species, containing at least seven DNA fragments (Figure 1B, ‘multimers,’ lanes 10–12). This band was not present in the absence of ligase, and thus could not be accounted for as an incompletely deproteinated sample (Figure 1B, lanes 7–9). This was further supported by the observation that the restriction site used to generate the initial fragments was reformed via ligation because treatment with the restriction enzyme used to generate the starting material led to the disappearance of the multimeric band (Supplementary Figure S1A). We characterized the multimeric band as DNA fragments ligated into a linear chain, as opposed to circular or catenated species, because spiking the gel with ethidium bromide, which induces supercoiling of circular products, did not alter the migration of the ligated material (28–31) (Supplementary Figure S1B). Also, in our experiment in Figure 1, we observed that the total number of DNA molecules ligated increased in the presence of MutLα, in addition to shifting the ligation products from smaller ligated species to larger multimeric fragments (Figure 1B). These data suggest that MutLα is promoting *inter-*molecular DNA associations.

The stimulation of DNA–DNA associations as measured by the formation of the multimeric product in the presence of MutLα was not specific to the polarity or sequence of the overhang. We found that when we used a 2.7 kb linear DNA fragment with a four nucleotide 3′-overhang, we generated the multimeric DNA product in a MutLα-dependent manner to a similar extent to when the fragments had 5′-overhangs (Supplementary Figure S1C). We also found that MutLα’s ability to facilitate *inter*-molecular associations was not dependent on the DNA sequence being ligated because 5′-overhangs of different sequences promoted identical results (Supplementary Figure S1D). These data suggest that the promotion of *inter*-molecular associations is likely topologically driven by the duplex region and not specific to the nature of the DNA ends.

We also wanted to determine whether MutLα could promote *intra*-molecular DNA associations which in mismatch repair could occur through DNA bending near the mismatch. To do this, we performed the ligation-based assay under conditions with low concentrations of DNA, so that the ligase will form a detectable amount of the circular (*intra*-molecular) product in addition to multimeric (*inter*-molecular) products (Figure 1C). By using a low concentration of DNA relative to the previous experiment, collisions between ends of the DNA molecule are more likely than collisions between distinct DNA molecules. Using these conditions, we observed a shift away from forming the *intra-*molecular circular product towards forming the *inter-*molecular multimer product as MutLα was titrated into the reaction (Figure 1D). These data suggest that MutLα has a higher tendency for associating distinct DNA molecules than it has for bending and promoting associations on the same DNA molecule. This could potentially be caused by a MutLα polymer stiffening the DNA helix to which it is bound and inhibiting bending at the site where the protein is bound. Cohesins and cohesin-like proteins, which facilitate DNA–DNA associations through a single protein binding to two DNA molecules, have displayed similar behavior in nearly identical assays (32,33). These experiments have been used to characterize and classify proteins as being cohesin-like and suggest that MutLα may have similar properties.

To determine if ligation of *inter-*molecular DNA species is driven explicitly by DNA affinity and the protein's ability to form oligomers, we took advantage of previous work illustrating that MutLα has higher affinity for larger substrates than smaller substrates (13). To do this, we performed the ligation-based assay described above with a 2.7 kb, low affinity, DNA substrate and compared it to a 4.3 kb, high affinity, substrate (Figure 1E). We generate both fragments with the same restriction enzyme and performed the assay under conditions where the same number of DNA ends were present, so the only difference between the reactions is the length of the DNA substrate. We found that while MutLα can promote ligated multimer formation for both the 2.7 and 4.3 kb substrates, multimer formation was somewhat more efficient on the lower-affinity, 2.7 kb substrate (Figure 1E). Using 300 nM MutLα, of the total ligated material ∼62% was multimerized for the 2.7 kb initial fragment relative to ∼54% for the 4.3 kb initial fragment (Figure 1E, lanes 6 and 10). This suggests that MutLα affinity and oligomerization alone are not responsible for promoting DNA end associations and an additional mechanism is required.

Based on these data, there are two potential models for how DNA–DNA associations could be facilitated in this assay (Figure 1F). MutLα could be promoting DNA associations either through a MutLα complex associating with two distinct DNA molecules simultaneously (Figure 1F, Model 1) or discrete MutLα-DNA complexes associating with one another through MutLα interactions (Figure 1F, Model 2). Based on our data, we favor Model 1 because in DNA binding experiments measuring affinity differences between linear DNA molecules of different sizes, when smaller DNA molecules are used, there are more free DNA molecules (i.e.—molecules that are completely devoid of MutLα proteins) than when larger DNA fragments are used (13). This indicates that in our experiment in Figure 1E, there are more free 2.7 kb fragments relative to the reactions containing the 4.3 kb fragments. Because multimerization of the 2.7 kb fragment was more efficient than the 4.3 kb fragment, even though the same number of DNA ends and the sequence of those ends were equivalent, this suggests that the multimerization is likely driven through interaction between a MutLα-DNA complex and a distinct DNA molecule.

### An excess of MutLα is required to tether DNA molecules

**Figure 2.**
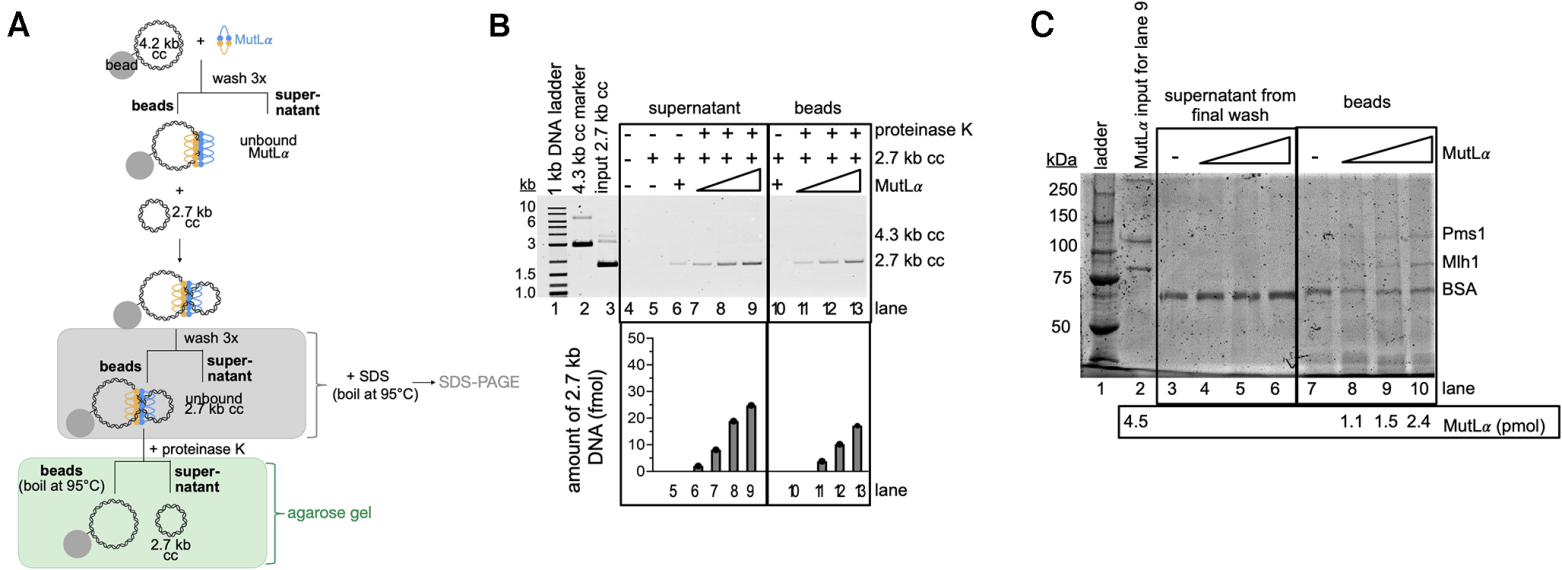
An excess of MutLα is required to promote DNA–DNA associations. (**A**) Schematic depicting DNA pull-down experiment. Biotinylated 4.3 kb covalently closed (cc) plasmid is immobilized on streptavidin beads. MutLα is bound to immobilized DNA and a 2.7 kb unbiotinylated plasmid is added to determine if the MutLα that is already bound to DNA can pull-down additional DNA molecules. Fractions that are analyzed by agarose gel are shown in green and fractions that are denatured and analyzed by SDS-PAGE are shown in grey. (**B**) Agarose gel assaying supernatant and bead-bound fractions. Lane 1 contains 1 kb plus DNA ladder (NEB), lane 2 contains a 4.3 kb DNA marker and lane contains DNA inputs for 2.7 kb (135 ng, 77 fmol) plasmids. In lanes 6 and 10, 8.0 pmol MutLα was added. In lanes 7–9 and 11–13, MutLα titration amounts are 2.6 pmol, 8 pmol, and 13.3 pmol respectively. Amount of recovered 2.7 kb DNA (fmol) relative to the input is shown in bar graph below the gel. Number of replicates is 2. (**C**) SDS-PAGE analysis of supernatant and bead-bound fractions. Lane 1 contains Precision Plus protein ladder (BioRad). Lane 2 contains the MutLα input for lane 9 (8.0 pmol). The Mlh1 subunit weighs 87 kDa and the Pms1 subunit weighs 99 kDa.

Despite our ligation experiments suggesting that MutLα oligomers and DNA affinity alone are insufficient to explain DNA–DNA associations, we wanted to confirm whether MutLα oligomers are necessary to promote this activity. To do this, we determined the stoichiometry of the protein and DNA components in a synapsed MutLα complex using a pull-down method to isolate the DNA–DNA associated complex formed by MutLα. To do this, we biotinylated a 4.3 kb circular substrate and immobilized it on streptavidin beads (Figure 2A). We removed unbound biotinylated DNA and incubated the immobilized DNA with MutLα. After washing away unbound MutLα, we added an unbiotinylated 2.7 kb circular substrate and incubated the material for 10 min at room temperature to allow the immobilized, DNA-bound MutLα complex to capture the second DNA molecule. We then washed away any unbound, mobile 2.7 kb DNA so that the only material in the sample is the bead-bound 4.3 kb DNA, any bound MutLα, and any 2.7 kb DNA that is immobilized via DNA–DNA associations promoted by MutLα. We then treated the sample with proteinase K so that we could measure the amount of 2.7 kb DNA pulled down via the synapsed complex.

Upon analyzing the supernatant after proteinase K treatment, along with the bead-bound fractions, we determined that MutLα restrained to immobilized DNA could indeed pull-down additional unbiotinylated DNA (Figure 2B, lanes 7–9). In the absence of MutLα, only background 2.7 kb DNA was detected (Figure 2B, lane 5). In the presence of MutLα, the 2.7 kb substrate was retained on the beads and was dependent on the concentration of MutLα that was bound to the immobilized plasmid (Figure 2B, lanes 7–9). These data are consistent with a DNA-bound MutLα complex associating with a second DNA molecule and are consistent with experiments measuring an analogous mechanism for cohesin proteins using a similar assay (34,35). It should be noted that our denaturing steps were insufficient to dissociate the biotinylated 4.3 kb substrate from the streptavidin beads due to the near-covalent affinity of the interaction. It should also be noted that we were unable to quantitatively recover the 2.7 kb plasmid as our proteinase K digest was incomplete. Even after digestion, the 2.7 kb substrate was retained on the beads and only removed by a further denaturing step necessitating boiling for 5 min at 95°C prior to electrophoresis which allowed us to remove the remaining 2.7 kb substrate from the beads (Figure 2B, lanes 11–13). This suggests that the MutLα-DNA complex may be compacted and in a conformation that at least partially protects MutLα from protease digestion.

We used SDS-PAGE to measure the amount of MutLα protein in the synapsed complex associating an immobilized 4.3 kb DNA to a 2.7 kb DNA. As depicted in the schematic in Figure 2A, we isolated the bead bound 4.3 kb DNA and the associated MutLα and 2.7 kb plasmid. We treated this sample and the supernatant from the final wash to remove unbound material with SDS and boiled at 95°C for 5 min. We then analyzed this material by SDS-PAGE (Figure 2C). For the bead bound 4.3 kb DNA and the associated MutLα and 2.7 kb plasmid, we recovered a stoichiometric excess of MutLα compared to DNA (Figure 2C, lanes 8–10) (∼100:1), suggesting that a large MutLα complex is required to simultaneously interact with two DNA molecules. A similar stoichiometric ratio is required to activate the endonuclease function of the enzyme (4,25,36). As controls, the samples from the final wash step did not contain detectable amounts of MutLα (Figure 2C, lanes 4–6). This suggests that MutLα is not dissociating to a high degree throughout the experiment and that the 2.7 kb DNA is pulled down through binding to a MutLα oligomeric complex that is also associated with the bead-bound 4.3 kb DNA.

Our data indicate that the MutLα-DNA complex that initially forms, facilitates DNA–DNA associations. Although we cannot completely characterize the nature of the initial MutLα–DNA complex and identify whether all of the MutLα proteins in the complex are directly associated with DNA, our data suggest the ability of a DNA-associated MutLα complex to bind to a second DNA molecule. This is consistent with work initially proposed by Hall *et al.* (13).

### *Inter*-molecular interactions activate of MutLα’s endonuclease activity

**Figure 3.**
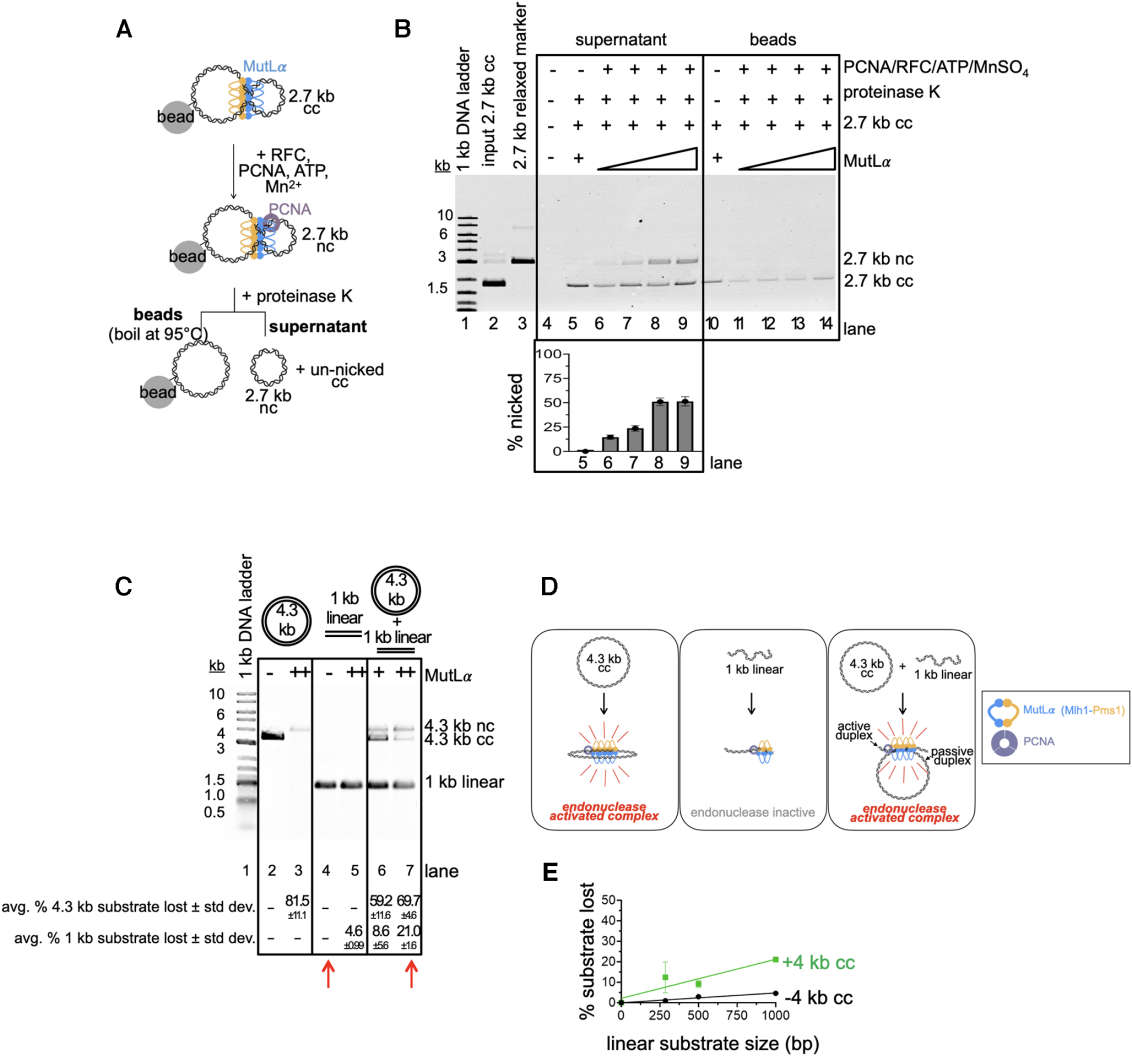
DNA tethering by MutLα promotes endonuclease activity. (**A**) Schematic depicting addition of endonuclease activating factors to DNA tethered by MutLα. (**B**) Native agarose gel assaying nicked 2.7 kb DNA from supernatant and bead-bound fractions. Lane 1 contains 1 kb plus DNA ladder (NEB), Lane 2 contains DNA input for 2.7 kb (135 ng, 77 fmol) plasmids and lane 3 contains 2.7 kb relaxed circular marker. For lane 5, MutLα concentration is 4 pmol and for lanes 6–9 MutLα concentrations are 2, 3, 4 and 6 pmol respectively combined with 10 pmol PCNA, 2 pmol RFC, 2.5 mM MnSO4, and 0.25 mM ATP. Lanes 11–14 are from boiled bead samples containing same MutLα titration. After addition of the endonuclease activating components, reactions were incubated at 37°C for 60 min prior to addition of protease and separation of supernatant and bead-bound fractions. The amount of DNA that is nicked is measured as the band intensity in the nc position relative to sum of the intensity in the nc band and the cc band. (**C**) MutLα can be promoted to nick a 1 kb linear substrate that is not nicked in isolation, in the presence of a second, larger (4.3 kb circular) DNA substrate. The 4.3 kb substrate is commercially available pBR322 (Invitrogen) and the 1 kb substrate was generated by isolating PCR products of these sizes. For both the 1 kb and 4.3 kb substrates, the final nucleotide concentration is 20 μM. MutLα was present at either 100 nM (+) or 200 nM (++) final concentrations. Endonuclease products are analyzed by denaturing gel as described in the Materials and Methods. Endonuclease activity is quantified as a loss of signal in substrate band relative to negative controls. Data below the gel represent the average and standard deviation of 3 experiments. Red arrows indicate lanes that should be compared to observe nicking in trans activity of the 1 kb fragment. (**D**) Model for MutLα activity in assay in panel C. RFC was also included in the reaction to load PCNA, but is not depicted. (**E**) Experiments performed identical to panel C at the 200 nM MutLα titration point. Gels were quantified and plotted based as the signal lost of the smaller DNA fragment in a denaturing agarose gel both with and without the 4.3 kb DNA. Experiment was performed in triplicate and error bars represent standard deviation between experiments.

In mismatch repair, a critical step in the initiation of the pathway is the generation of a nick by MutLα. We wanted to determine whether *inter*-molecular DNA associations could stimulate the MutLα endonuclease. In Figure 2, we were able to characterize the MutLα complex that is promoting DNA–DNA associations. We next tested whether this complex was indeed endonuclease active. Using our tethering experiment in Figure 2, after pulling down the unbiotinylated 2.7 kb plasmid with MutLα bound to biotinylated 4.3 kb DNA, we added the MutLα-activating factor PCNA, along with its loader RFC, ATP and Mn^2+^ to stimulate the endonuclease activity (Figure 3A). After incubating the pulled-down material with the additional reaction components, we assayed the tethered, unbiotinylated 2.7 kb circular DNA and found that the DNA was nicked efficiently (Figure 3B). We found that as we added increasing amounts of MutLα to the reaction, increasing amounts of the 2.7 kb circular DNA was nicked until a maximum activity is reached (Figure 3B, lanes 6–9).

Having observed that the MutLα complex that we pulled down with an immobilized DNA is endonuclease active and that at least some of its activity is directed to the mobile 2.7 kb DNA that was pulled down, we wanted to test whether the tethering activity stimulates endonuclease activity. We performed an experiment analogous to that previously reported by Manhart *et al.* where the endonuclease activity of MutLγ could be activated on a small DNA substrate that did not support the activity in isolation in the presence of a larger substrate that supports endonuclease activity robustly (14). To identify a substrate to serve as the small DNA molecule that does not support MutLα endonuclease activity by itself in this assay, we generated linear substrates ranging in size from 4.3 kb to 286 bp. In reactions containing equivalent amounts of total nucleotide, we measured MutLα endonuclease activity and found that although larger DNA substrates were preferred, we could still detect nonspecific endonuclease activity on substrates as small as 2 kb when analyzed by denaturing gel (Supplementary Figure S2A, B). We observe endonuclease activity at approximately background levels on substrates ≤1 kb. We could not induce activity on these substrates even with incubation times up to 24 h (Supplementary Figure S2C). These data are consistent with work previously demonstrating that MutL homologs have higher affinity and are more endonuclease active on larger DNA substrates and the oligomeric nature of the endonuclease (13–15,21).

We next assayed for endonuclease activity of substrates that are not nicked in isolation in the presence of a 4.3 kb circular substrate that is nicked efficiently by itself in the presence of 200 nM MutLα (Figure 3C, lane 3). In the presence of this larger, circular substrate, we were able to stimulate the endonuclease activity on the 1 kb linear substrate in *trans* (Figure 3D). In the absence of the 4.3 kb circular substrate, in reactions containing 200 nM MutLα, we could only observe ∼5% of the substrate being nicked. In the presence of the 4.3 kb circular DNA, we could increase the nicking efficiency to ∼20% (Figure 3C, compare lane 4 with lane 7, red arrows) using this same amount of protein. If nicking was exclusively driven by the affinity of MutLα for DNA, then we would expect that addition of large DNA molecules to reactions with small substrates that do not support endonuclease activity in isolation, would have no effect, because the protein would preferentially bind to the higher affinity DNA molecules. If anything, the larger DNA molecules would have an inhibitory effect by titrating MutLα away from the smaller fragments. In our assay, however, we are able to activate endonuclease activity on small fragments in the presence of large, high affinity DNA molecules. In these reactions, endonuclease activity appears to be titrated away from the 4.3 kb circular substrate by the lower affinity, 1 kb linear fragment (Figure 3C, compare lane 3 with lane 7). These data suggest that MutLα tethering two distinct DNA molecules can stimulate endonuclease activity. It also suggests that although tethered to both DNA substrates, the MutLα complex may only be nicking one duplex—interacting with one DNA duplex passively and another duplex actively.

Together, our data suggest that *inter*-molecular DNA interactions driven by a MutLα oligomeric complex stimulate endonuclease activity. Although modest, we were also able to stimulate activity on the 500 and 286 bp fragments in the presence of the 4.3 kb circular DNA to levels significantly higher than in the absence of the larger DNA (Figure 3E). These data also suggest that an endonuclease active MutLα complex requires ≤286 bp of DNA to form. In these assays, larger linear substrates nicked in *trans* are nicked more efficiently than smaller substrates nicked in *trans* (Figure 3E, compare sizes on green line). The observation that there is a size dependence for this nicking in *trans* activity, is analogous to previous data demonstrating a dependence between substrate size and affinity which was first used to propose MutLα oligomers (13), and suggests that although insufficient on its own to explain the endonuclease activation, oligomerization may be necessary for stimulation of the enzyme.

If MutLα acts as a cooperative, oligomeric enzyme, a larger DNA molecule would more efficiently support the required amount of protein to activate the endonuclease activity. Smaller substrates do not efficiently accommodate sufficient MutLα oligomers to induce endonuclease activity, explaining the near zero nicking efficiency for small DNA substrates in single substrate reactions (Supplementary Figure S2A, B). Once a substrate reaches a length where it can support a critical number of MutLα proteins, a near linear dependence on size is observed until the amount of protein is limiting relative to DNA and the relationship saturates (Supplementary Figure S2B). If interactions between two DNA molecules are also required to support MutLα endonuclease activity, our observations on the single substrate reactions using small linear substrates can be explained by a model where MutLα does not have a large enough expanse of DNA between ends to form a stable oligomer without sliding and dissociating from the ends. On larger substrates, a MutLα oligomer has more initiation sites distant from ends and is more able to form an oligomer of sufficient length before encountering an end and sliding off. Once the oligomer is formed, it can collide with other DNA molecules in the reaction to activate the endonuclease activity. In the experiments in Figure 3C–E, the presence of the 4 kb circular substrate could restrain and stabilize a MutLα oligomer so that it can collide with the substrates that are 1 kb and smaller to promote activity.

### DNA topology influences the activation of the MutLα endonuclease

The preference of MutL proteins for larger substrates has previously been attributed to the cooperative nature of MutL binding and the ability of larger DNA to support more MutL proteins (13,14). It has been suggested that circular substrates are preferred relative to linear topologies because circular DNA molecules do not allow MutL proteins to slide off of the ends (14). An alternative, non-mutually exclusive model, is that tethering of two duplex regions by a MutLα oligomeric complex is necessary to activate endonuclease function. Such a model accounts for increased activity on larger, more flexible substrates and a preference for circular substrates which have inherent curvature positioning adjacent regions of duplex in relatively close proximity. A strand capture mechanism for activation on linear DNA would require either collision with another linear molecule or bending of the linear DNA by a MutLα polymer which are inefficient and could account for the decreased activity on this topology.

**Figure 4.**
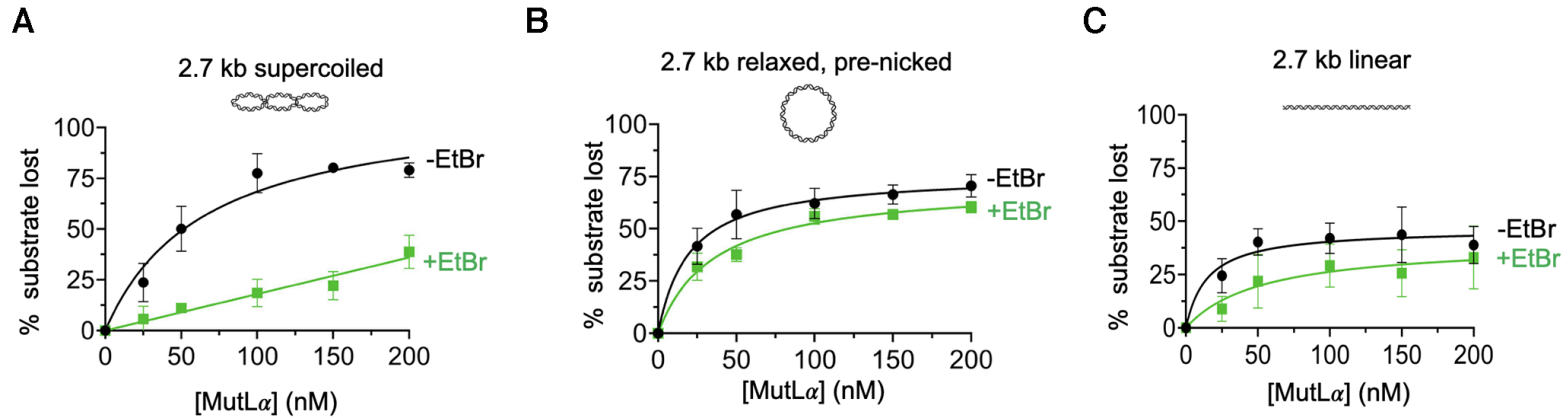
Activation of the MutLα endonuclease must overcome DNA supercoiling. (**A**) MutLα endonuclease activity on supercoiled, closed circular pUC18 DNA substrate is inhibited in the presence of ethidium bromide (EtBr) intercalator. MutLα is included in reactions at 25, 50, 100, 150 and 200 nM final concentrations. RFC and PCNA are included at 100 nM and 500 nM, respectively along with 2.5 mM MnSO4 and 0.25 mM ATP. Where included, ethidium bromide is present at 5 μg/ml. Experiments were resolved by agarose gel and quantified data is reported here for reactions with (green squares) and without (black circles) ethidium bromide (EtBr). Number of replicates are 4. Data points represent averages and error bars indicate standard deviation between experiments. Graph was fit to hyperbolic model. (**B**) MutLα endonuclease activity on 2.7 kb pre-nicked DNA substrate (Nt.BspQI) in the presence (green squares) and absence (black circles) of ethidium bromide intercalator. The experiment is identical to that in panel A. Data points represent the average of three replicates. (**C**) MutLα endonuclease activity on 2.7 kb linear substrate generated by HindIII digestion in the presence (green squares) and absence (black circles) of ethidium bromide intercalator. The experiment is identical to that in panels A and B. Data points represent the average of three replicates.

We further tested the role of DNA topology for the activation of the MutLα endonuclease. We measured the endonuclease activity of MutLα on commercially available 2.7 kb substrate, where >90% of the molecules are in a supercoiled conformation (Figure 4A, black line). To test for effects of DNA topology, we compared this activity to activities on equivalent circular DNA that is relaxed by the presence of a single pre-existing nick (Figure 4B, black line). Past work has indicated that supercoiled and relaxed variants of the same plasmid are bound with near equal affinities (13). Our data demonstrating that MutLα complexes can simultaneously associate with multiple DNA regions and that this property can activate the endonuclease suggests that supercoiled DNA molecules may be more efficient substrates than identically sized relaxed plasmids because supercoiled DNA consists of regions of DNA in close proximity whereas relaxed DNA would need to be bent in order to facilitate DNA–DNA interactions. Consistent with this, we found that nuclease activity was moderately higher on supercoiled DNA relative to relaxed at high concentrations of MutLα. For example, using 150 nM MutLα, ∼80% of the supercoiled DNA is nicked compared to ∼66% of the relaxed plasmid. Surprisingly, using lower concentrations of MutLα, nicking is more efficient on the relaxed plasmid than on the supercoiled counterpart. For instance, when 25 nM MutLα is included in the reaction, ∼24% of the supercoiled DNA is nicked compared to ∼42% of the relaxed plasmid. It should be noted that the supercoiled and relaxed substrates are of identical size and sequence and in past work, have been shown to bind to MutLα with near identical affinities (13). The observation that they behave differently from one another in endonuclease assays is consistent with our experiments in Figure 3, which suggest that an additional mechanism besides MutLα oligomer binding on DNA is likely necessary for endonuclease activation.

The observation that relaxed circular DNA substrate was preferred at low concentrations of MutLα over supercoiled suggests that a MutLα complex may use its ability to associate two DNA regions to alter DNA topology into a particular conformation to activate the enzyme. To test this, we added a planar ligand that acts as an intercalating agent, increasing the rigidity of the DNA polymer, in addition to inducing and stabilizing supercoiling (31,37–39). In the presence of ethidium bromide, MutLα’s nicking efficiency on supercoiled DNA was inhibited compared to reactions without the intercalator (Figure 4A, compare black and green lines, Supplementary Figure S3A). At the 200 nM titration point, the amount of DNA nicked in reactions with ethidium bromide was reduced ∼2-fold compared to reactions without the ligand. Similar results were observed in the presence of another DNA intercalator, acridine orange (Supplementary Figure S3B). On the relaxed, pre-nicked circle, addition of ethidium bromide was only mildly inhibitory. Using a final concentration of 200 nM MutLα, the amount of DNA nicked was reduced only by ∼1.2-fold in the presence of the intercalator (Figure 4B, compare black and green lines). The degree of inhibition of endonuclease activity on linear substrates was similar to that on the pre-nicked, relaxed circle (Figure 4C). Further supporting a topological role for DNA in activating the MutLα endonuclease, we observed that ethidium bromide was unable to inhibit sequence specific restriction endonucleases on supercoiled substrates (Supplementary Figure S3C).

**Figure 5.**
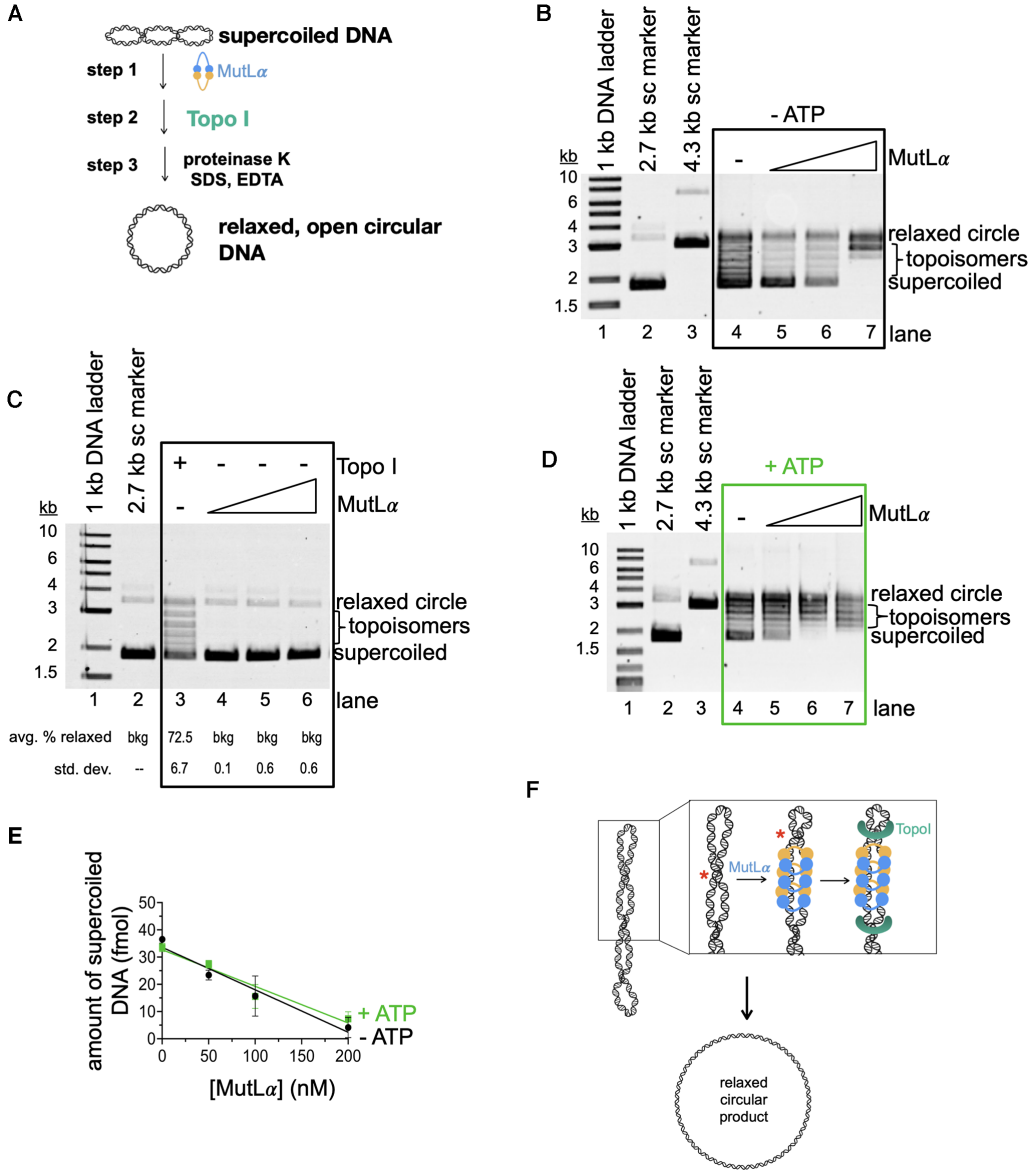
MutLα stimulates DNA relaxation by a topoisomerase *in vitro*. (**A**) Schematic depicting topoisomerase assay. Supercoiled pUC18 DNA was incubated with MutLα (step 1) followed by addition of E. coli Topoisomerase I (step 2) which converts supercoiled DNA into relaxed, open circular DNA. The protein component of reactions is degraded and the DNA end products are analyzed by native, ethidium bromide stained agarose gel. (**B**) Agarose gel depicting DNA relaxation by Topo I and MutLα in the absence of ATP. Lane 1 contains 1 kb plus DNA marker and lanes 2 and 3 contain 2.7 and 4.3 kb supercoiled (sc) DNA markers respectively. Lane 4 is a negative control without MutLα and lanes 5–7 contain a titration of MutLα (50, 100 and 200 nM). (**C**) Agarose gel depicting MutLα activities in this assay in the absence of Topo1. Lane 3 is a positive control with Topo1. In lanes 4–6, MutLα was titrated at 50, 100 and 200 nM final concentrations. Number of replicates is 3. (**D**) Experiment and gel are identical to those in panel B, but in the presence of 0.25 mM ATP. (**E**) Plot for gels in B and D showing amount of supercoiled DNA versus MutLα concentration. Black circles are conditions without ATP and green squares are conditions with ATP. Experiment was performed in triplicate. Data was fit to a simple linear regression curve with error bars representing the standard deviation between experiments. (**F**) Model for MutLα activities in this assay. Red asterisk indicates the repositioning of the helical crossover.

Because ethidium bromide had a more pronounced inhibitory effect on MutLα endonuclease activity on the covalently closed DNA capable of adopting a supercoiled conformation, we hypothesized that MutLα may alter the topology of supercoiled DNA. To test this, we performed an assay using Topoisomerase I (Topo I) from *E. coli*, which catalyzes the relaxation of supercoiled DNA in an ATP-independent mechanism (Figure 5A). In this assay, we first incubated MutLα with supercoiled DNA using conditions that promote DNA binding, but not endonuclease activity (i.e. PCNA and MnSO_4_ were omitted for all reactions). We then, added Topo I at an amount where ∼45% of supercoiled DNA is converted to relaxed topoisomers (Supplementary Figure S4). After incubation with Topo I, we deproteinate that reaction so that the DNA end products can be analyzed in the absence of bound protein. We found that as we added increasing amounts of MutLα, we could increase the amount of relaxed topoisomers relative to reactions without MutLα (Figure 5B). At concentrations of MutLα that support maximum endonuclease activity on this substrate in the presence of PCNA and MnSO_4_, 200 nM, we increase the yield of the Topo I reaction by ∼2-fold relative to reactions without MutLα. The stimulation of the topoisomerase is not due to MutLα nicking the DNA as indicated by control experiments performed in the absence of the topoisomerase (Figure 5C). In these reactions, the amount of relaxed product in the presence of just MutLα is equivalent to negative controls without MutLα or Topo I. These data suggest that MutLα is altering DNA topology to aid the topoisomerase without the use of its endonuclease activity (Figure 5F).

In addition to MutLα being an endonuclease, it also possesses ATPase activity in each subunit of the heterodimer (40). Previous work has shown that the protein's conformation is modulated by ATP occupancy and that conformational changes modulate DNA binding and endonuclease activities (3,4,25,41–45). We wanted to determine if MutLα’s ATPase function regulates its ability to stimulate a topoisomerase. To test this, we performed an experiment identical to that described above but also included ATP in step 1 with MutLα (Figure 5A). We found that when ATP was added, the total effect of MutLα on DNA relaxation by Topo I was comparable to reactions without ATP, as measured as a loss of supercoiled DNA (Figure 5D, E). We found that in the absence of ATP, most of the DNA was either found in the supercoiled or completely relaxed band position. In the presence of ATP, we observed that more DNA was found in semi-relaxed topoisomers as opposed to the completely relaxed species (compare topoisomer bands in gels in Figure 5B and D). This suggests that in the presence of ATP, although MutLα still enhances Topo I efficiency overall, it may be less effective at partially relaxing regions of supercoiled DNA. This is likely accounted for past work demonstrating that MutLα has less affinity for DNA in the presence of ATP (25,41,42,45).

## DISCUSSION

Here, we observed that the primary DNA mismatch repair endonuclease, MutLα, facilitates DNA associations driven by topological DNA binding, which in turn, promotes endonuclease function. We also determined that MutLα complexes affect DNA supercoiling without using endonuclease activity. Our work uses DNA ligation assays and pull-down experiments to characterize the MutLα polymer as well as suggest that mismatch repair proteins can promote simultaneous association with distinct DNA duplexes. We hypothesize that this property serves to activate the endonuclease activity of the protein and that DNA affinity alone is insufficient to describe endonuclease activation. Our data suggest that MutLα can alter DNA topology by measuring enhancement of a topoisomerase that relaxes supercoiled plasmid. Together, these activities may serve to restrain DNA nicking to a localized region of mismatched DNA and may facilitate access to the DNA near the mismatch by other factors. Additionally, our data also suggest how MutL proteins may act in other pathways, such as DNA transaction processes.

Previous work has shown that multiple MutLα proteins are present on DNA during mismatch repair (12,15,18,46) and that MutLα binds to DNA in a cooperative manner (13). This property is shared by other MutL homologs, and is required to activate the endonuclease activity (14). Our work suggests that an additional role for MutLα cooperativity is to facilitate associations between DNA duplex regions which are also necessary for triggering endonuclease function. We saw evidence that yeast MutLα promotes interactions between linear DNA fragments through a tethering mechanism with our ligation assays (Figure 1) and in pull-down experiments (Figure 2). We observed that associations between DNA duplex regions may be important for MutLα function because we were able to observe endonuclease activity on tethered DNA substrate (Figure 3). These data suggest that a DNA duplex-bound MutLα complex may need to capture a second duplex to become activated.

Although we cannot definitively determine whether each MutLα in this initial complex is simultaneously bound to two helices, our observations are reminiscent of work characterizing other proteins that facilitate DNA interactions. Past work has indicated that prokaryotic cohesin-like protein RecN, as well as other cohesins, promote *inter*-molecular DNA associations via DNA tethering accompanying topological binding (32–35). Strikingly, the type IB DNA topoisomerase (TopIB) has been shown in biophysical work to form a polymeric complex on large DNA substrates that facilitates DNA associations to stabilize binding to supercoils prior to relaxation (47–49). On circular substrates, TopIB can facilitate *intra*-molecular interactions between distant regions of DNA via two distinct DNA binding sites on a single TopIB protein (48). On linear substrates, TopIB primarily facilitates *inter*-molecular interactions between distinct linear substrates, likely due to the fact that collisions between DNA molecules are more frequent than bending (47,49). Such a model could explain why linear DNA molecules are nicked with less efficiency by MutLα than circular substrates. The topology of circular DNA forces additional DNA duplex in close proximity of a MutLα binding site whereas linear DNA requires collision. Although DNA binding sites in MutLα have been identified in the amino-terminal domains (50–52) and recent structural work suggests residues in the amino terminal domain of *E. coli* MutL that interact with a primer-terminus (27), follow-on work is needed to determine if there are additional sites and to explicitly measure synergy between the known sites. Additional work is also required to understand how individual MutLα proteins interact with one another in an oligomeric complex.

**Figure 6.**
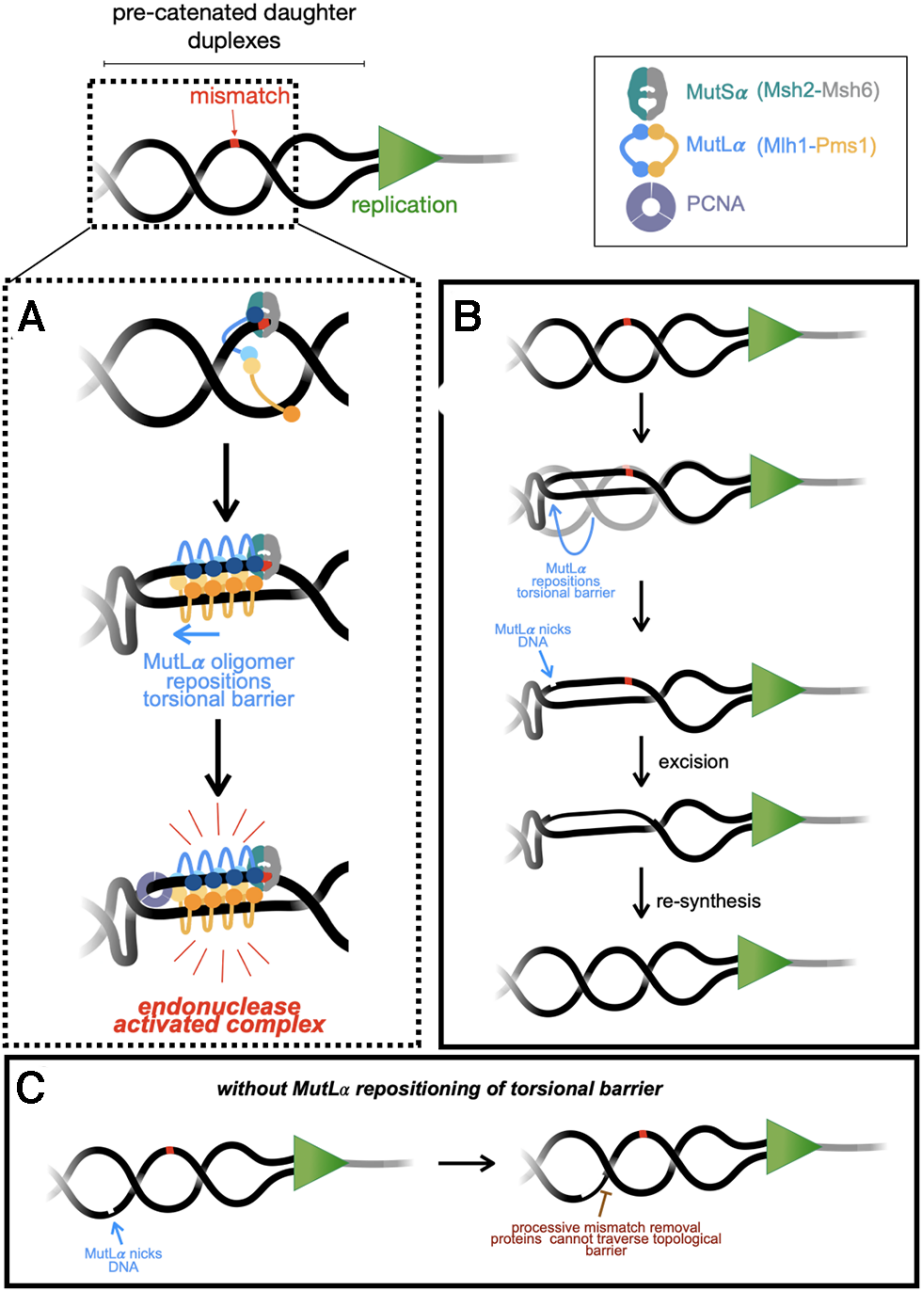
Model for MutLα activities in mismatch repair. (**A**) MutLα is recruited to topologically complex DNA near a mismatch (red) by a MutS homolog (shown here as MutSα). MutLα proteins form an oligomer which can associate the two daughter DNA duplexes and reposition helical crossovers that can form behind the replication fork. MutLα is activated through the DNA–DNA association and via interaction with PCNA that is either loaded near the mismatch site or left behind by the replication machinery. MutLα alters the DNA topology in the vicinity of the mismatch through facilitating interaction with two duplex regions, one actively (which it will nick) and the other passively. (**B**, **C**) Repositioning of the topological barrier allows a long tract of DNA to be available for processive components of the DNA mismatch repair process that may be inhibited by topological barriers. See Discussion for additional details.

Our data investigating MutLα endonuclease activity in *trans* (Figure 3C–E) suggests that an endonuclease active tethered complex is likely smaller than 286 bp. This is consistent with previous experiments partially reconstituting MutLα activities in the presence of a mismatch and MutSα (3,4). In these experiments, MutLα nicks DNA within ∼100–200 bp of a mismatch on the opposing side of that mismatch from a pre-existing nick presumably used to load PCNA. Our data demonstrating a tethered, functional complex on a 286 bp substrate is also consistent with work in Bradford *et al.* where specific MutSα-MutLα complexes formed near mismatches and occluded ∼150–250 bp (18). Additionally, our data in Figure 3E supports an activated MutLα complex interacting with two duplex regions simultaneously, likely only in position to nick one duplex, suggesting that MutLα is interacting with one DNA duplex actively (Figure 6, daughter duplex with the mismatch) and another passively (Figure 6, other daughter duplex). Such a model could also account for data presented by Hall, *et al.*, showing that supercoiled DNA is bound by MutLα with higher affinity than equivalent linear or nicked circular DNA (13) and data demonstrating that MutLα incises mismatch-containing supercoiled DNA more effectively than mismatch-containing relaxed DNA in a partially reconstituted system (53). This is also consistent with our data in Figure 4A-B, suggesting that supercoiled DNA is nicked inefficiently at low concentrations of MutLα, because an adequate polymer is not formed to alter the DNA topology to activate the endonuclease.

*In vivo*, the major eukaryotic mismatch repair pathway is initiated via mismatch recognition by MutSα or MutSβ, followed by recruitment of MutLα. DNA near the mismatch, behind the replication fork, may present a unique torsional landscape that the repair machinery downstream of mismatch recognition must overcome. During DNA replication, relaxation of topological stress ahead of the replication machinery via fork rotation creates compensatory DNA intertwines or precatenanes behind the fork where mismatch repair machinery is acting in post-replicative mismatch repair (12,54,55). Fork rotation occurs with higher frequency in regions with repetitive sequences which are difficult to replicate and are prone to generating mismatches (56,57), suggesting that mismatch repair proteins may need to navigate precatenanes during the repair process. Helical crossovers in the precatenane structure could act as steric blocks for proteins that either diffuse along a helix (such as PCNA) or act processively (such as Exo1 or polymerases), potentially preventing them from accessing DNA near the mismatch to complete repair. We propose a model where upon recruitment by a mismatch-bound MutSα, MutLα partially alters the DNA topology near the mismatch through its DNA tethering activity (Figure 6A, B). Formation of a MutLα oligomer could aid in this process which also may serve as an activation mechanism for the endonuclease. MutLα oligomers have previously been described as being necessary to support endonuclease activity (14), but work here suggests that they may also be critical for altering DNA topology near mismatches to reposition helical crossovers, creating a tract between the nicking and mismatched site that is clear of topological barriers. Together, these activities could constrain nicking to a localized region of the DNA in the vicinity of the mismatch and can also remove obstacles for other repair factors, such as PCNA, which needs to interact with MutLα to activate endonuclease (3,4). It is not clear if the PCNA that activates MutLα in mismatch repair is left behind by the replication fork or is loaded during repair *de novo*. If loaded for post-replicative repair, MutLα’s ability to alter DNA topology may also create a landing site for RFC to load PCNA (53,58), although either model may result in topological challenges for PCNA to gain access to MutLα.

MutLα oligomers may also alter DNA topology so that downstream removal and resynthesis factors can access the DNA spanning the distance from the MutLα nick to the mismatch, which can be hundreds of base pairs away, as measured *in vitro*, without topological barriers (3,4,15). Removal of the mismatch from a MutLα-generated nick proceeds through the exonuclease activity of Exo1 or the strand displacement activity of polymerase δ, both of which function on DNA processively. If a topological barrier exists between the mismatch and the nick initiating repair, the mismatch may not be removed efficiently. Additionally, if MutLα repositions the helical crossover to the opposing side of the mismatch from the nick, it may serve a regulatory role in terminating excision and replication (Figure 6B, C).

In addition to their critical role in DNA mismatch repair, MutL homologs are involved in DNA transaction processes. In double strand break repair, DNA breaks are resected, creating single-stranded DNA tails which can invade duplex DNA. The invading strand can be extended by replication proteins, using the donor chromosome as a template. This is followed by second-end capture and formation of double Holliday junction substrates (59–64). In meiosis, MutL homologs MutLβ and MutLγ, which have minor roles in mismatch repair, participate in this process (65–67). MutLβ has been suggested to have an accessory role in DNA mismatch repair (68), but in meiotic recombination, it serves to limit extension of single-end invasion intermediates (69). MutLγ acts as a minor or backup endonuclease in DNA mismatch repair downstream of mismatches recognized by MutSβ (68), but in meiotic recombination, it acts as a non-canonical Holliday junction resolvase responsible for generating the majority of chromosomal crossovers (26,65,68,70,71). Much of our understanding of the roles of MutLβ and MutLγ in recombination is garnered from biochemical studies of MutL homologs in mismatch repair.

Recombination intermediates are branched in nature and result in positioning duplex regions of DNA from both the chromosome with the initiating double strand break and the donor chromosome in proximity to one another (63,72). Our work suggests that MutL proteins have the ability to tether duplex regions of DNA together. These activities are consistent with a cohesin-type tethering mechanism where proteins physically link homologous chromosomes. An appealing model involves MutL homologs encircling two duplex regions of DNA during recombination and altering the topology of these substrates to either limit replication in the case of MutLβ or nick Holliday junctions in orientations that promote crossing over in the case of MutLγ. Follow-on studies are necessary to directly determine whether other MutL homologs can simultaneously interact with two DNA molecules and whether this can occur on model recombination substrates. Past work has demonstrated that yeast MutLγ hydrolyzes circular DNA more efficiently than equivalent size and sequence linear DNA and can be promoted to nick small linear substrates in the presence of large circular substrates (14). These data are consistent with DNA tethering being a conserved property of MutL homologs.

## DATA AVAILABILITY

This study includes no data deposited in external repositories.

## Supplementary Material

gkad096_Supplemental_File
