# Supplementary material for: The mismatch repair endonuclease MutLα tethers duplex regions of DNA together and relieves DNA torsional tension
Source: Nucleic Acids Res. 2023 Feb 25;51(6):2725–39. doi: 10.1093/nar/gkad096 (PMC10085691; doi:10.1093/nar/gkad096)
Supplement: gkad096_Supplemental_File [file gkad096_supplemental_file.pdf]

SUPPLEMENTARY INFORMATION

Witte, et al., The mismatch repair endonuclease MutLα tethers duplex regions of DNA together and relieves DNA torsional tension

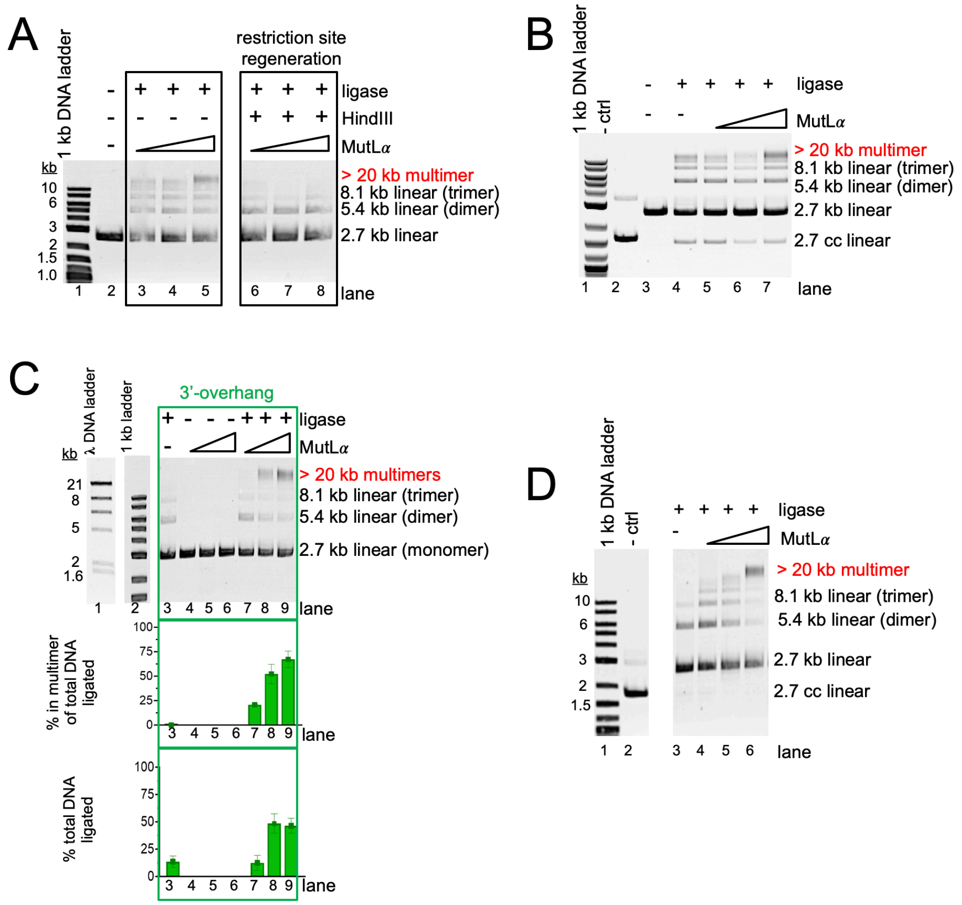

Figure S1. Characterization of MutLα-induced end-to-end DNA ligation product. (A) Ligation assay using 2.7 kb linear substrate (HindIII) and testing the reformation of the restriction site post ligation. Lanes 3-5 show normal ligation with MutLα titration of 50, 150 and 300 nM. Lanes 6-8 have same MutLα titration conditions but are incubated with 10 units of HindIII. All lanes were run on the same gel with intervening lanes removed for simplicity. (B) Ligation assay containing ethidium bromide in gel to characterize linear multimer versus circle formation. Ethidium bromide in gel was at final concentration of 0.5 μg/mL. Lanes 5-7 contain MutLα titration of 50, 150 and 300 nM. Lane 2 contains supercoiled 2.7 kb control and lane 3 contains linear 2.7 kb control for characterization. (C) Ligation assay as in Figure 1A. Linear 2.7 kb DNA with 3'-overhangs generated with the SphI restriction enzyme. Reactions were run in the same gel as in Figure 1B. Lanes 1 and 2 (also in Figure 1B) contain DNA markers for determining the size of the ligated products. Lambda DNA (NEB) marker was digested with HindIII. Where +, T4 DNA ligase is added as indicated in the Materials and Methods. In lanes where a titration of MutLα is indicated the concentration of protein is 50, 150 and 300 nM. All lanes were run on the same gel with material in Figure 1A, with intervening lanes removed for simplicity. The amount of signal in the highest molecular weight band was quantified relative to the total amount of ligated product in the gel and the amount ligated was quantified relative to total amount of DNA in lane. These are expressed in bar graphs below the gel. The experiment was conducted in triplicate and error bars represent standard deviations between experiments. (D) Ligation of linearized 2.7 kb DNA with different 5' overhang sequence (BsaI digested). In lanes where a titration of MutLα is indicated the concentration of protein is 50, 150 and 300 nM. All lanes were run on the same gel with intervening lanes removed for simplicity.

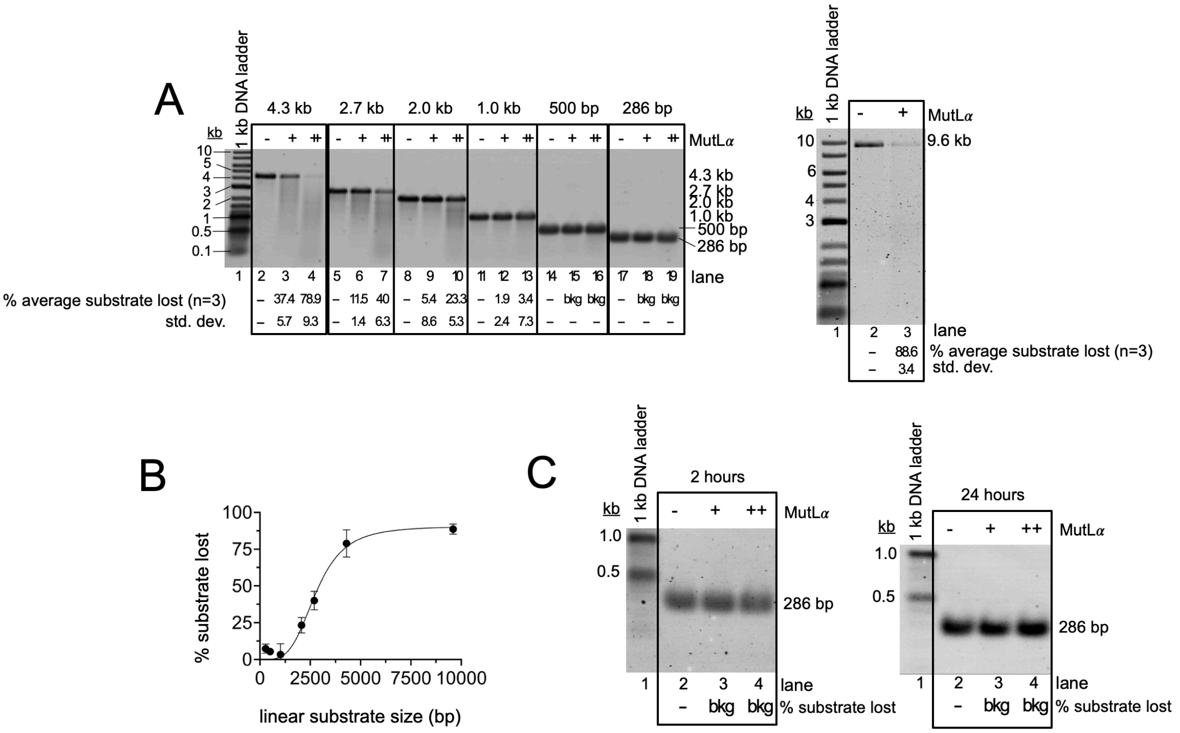

Figure S2. (A) Endonuclease assay measuring MutLα nicking efficiency on linear substrates of varying size. 0 nM (-), 20 nM (+), or 200 nM (++) MutLα was combined with 300 nM PCNA, 100 nM RFC, 2.5 mM $MnSO_4$, 0.25 mM ATP, and 20 μM (final nucleotide concentration) for each substrate. Activity was assayed by denaturing agarose gel as described in the Materials and Methods. The 4.3 kb and 2.7 kb linear substrates were generated by linearizing pBR322 or pUC18, respectively, with SspI according to the manufacturer's (NEB) instructions. The 2.0 kb substrate was generated by digesting pUC18 with SfoI and XmnI according to the manufacturer's (NEB) instructions and gel isolating the 2.0 kb fragment. The 1.0 kb, 500 bp, and 286 bp substrates were generated by isolating PCR products of these sizes. The 9.6 kb substrate was generated by linearizing 9.6 kb circular plasmid with EcoRI and was run on a separate gel to ensure appropriate resolution of the smaller fragments. (B) Quantification of the experiment shown in panel A at the 200 nM MutLα titration point. Data points are averages of three replicates. Error bars show standard deviation between experiments. (C) Endonuclease assay measuring MutLα nicking ability on same 286 bp substrate at longer incubation times.

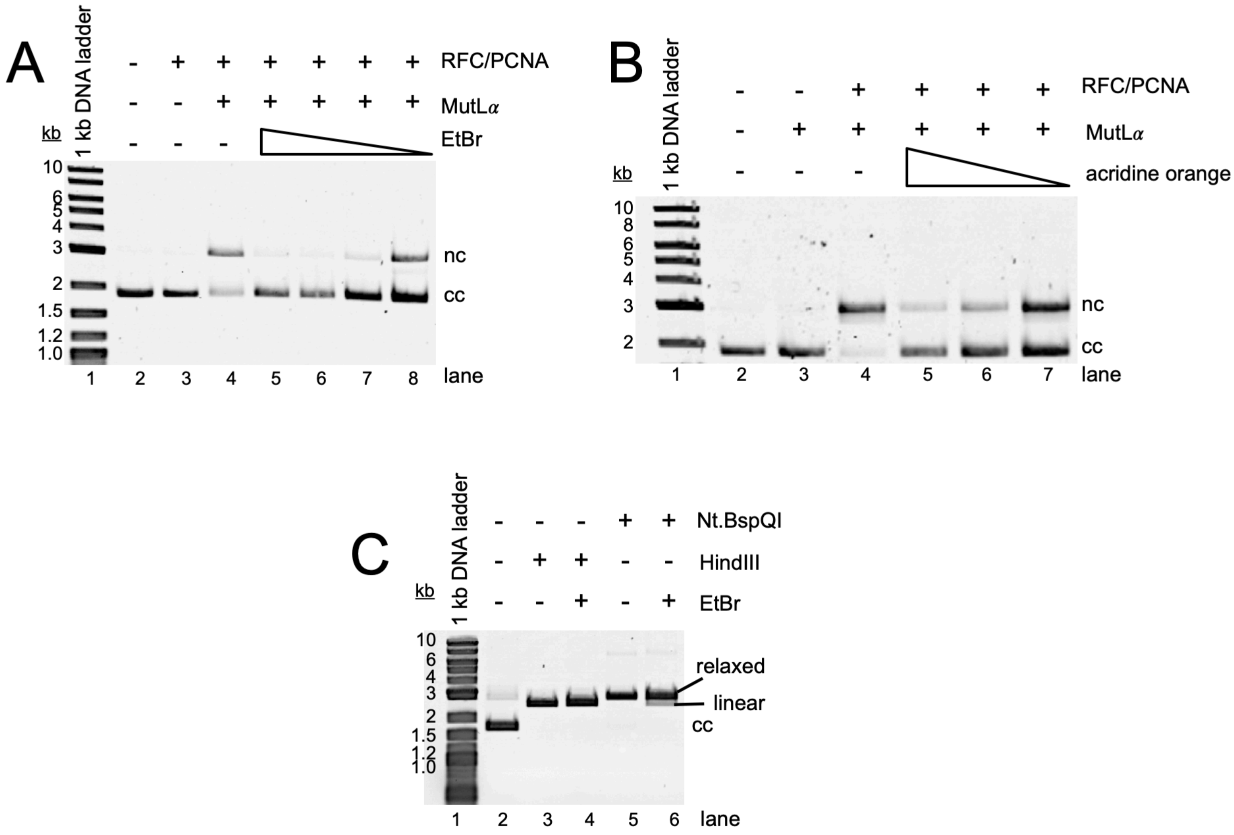

Figure S3. Stabilization of supercoiled DNA inhibits MutLα endonuclease activity. (A) Ethidium bromide inhibits endonuclease activity on circular substrate. For MutLα were + is indicated, the final concentration is 50 nM. For PCNA and RFC, where a + is indicated the final concentrations are 500 nM and 100 nM respectively. Lanes 5-8 contain a decreasing titration of ethidium bromide in the reactions. Starting at lane 5 the final concentration of EtBr is 0.25 mg/mL, lane 6 is 0.025 mg/mL, lane 7 is 2.5 μg/mL and lane 8 is 0.25 μg/mL (B) Acridine orange intercalator inhibits endonuclease activity on circular substrate. For MutLα*,* where + is indicated, the final concentration is 50 nM. For PCNA and RFC, where a + is indicated the final concentrations are 500 nM and 100 nM respectively. Acridine orange titration (lanes 5-7) are 0.025 mg/mL, 2.5 μg/mL and 0.25 μg/mL. (C) HindIII digestion and nicking by Nt.BspQI of circular substrate is not appreciably affected by ethidium bromide. 2.7 kb circular DNA substrate was added to a final concentration of 3.8 nM. Final concentration of EtBr is 5 μg/mL. For HindIII, 5 units were added to digest DNA and for Nt.BspQI, 2.5 units were added. Reactions were compared for complete digestion or nicking with control of circular 2.7 kb substrate in lane 2.

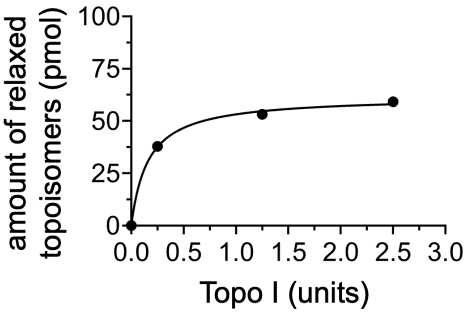

Figure S4. Titration of *E. coli* Topoisomerase I (Topo I) for Topoisomerase assays. Plot for optimization of *E. coli* Topoisomerase I on 2.7 kb supercoiled DNA. Amount of relaxed 2.7 kb DNA (fmol) was plotted vs units of Topo I (.25, 1.25 and 2.5 units). The data was fit to a hyperbolic model. The amount of relaxed DNA at 0.25 units (~37 pmol) is equivalent to ~48% relaxed.
